# Supplementary material for: Pyruvate Dehydrogenase Kinase 1 inhibition mediated oxidative phosphorylation enhancement in cartilage promotes osteoarthritis progression
Source: BMC Musculoskelet Disord. 2023 Jul 20;24:597. doi: 10.1186/s12891-023-06585-6 (PMC10357736; doi:10.1186/s12891-023-06585-6)
Supplement: Supplementary file 1 — Additional file 1. [file 12891_2023_6585_MOESM1_ESM.zip › BP.Gsea.1653623667859/GOBP_CYTOSKELETON_DEPENDENT_CYTOKINESIS.html]

Details for gene set GOBP\_CYTOSKELETON\_DEPENDENT\_CYTOKINESIS[GSEA]

|  || Dataset | 55457\_collapsed\_to\_symbols.cls.cls #OA\_versus\_normal.cls.cls #OA\_versus\_normal\_repos |
| Phenotype | cls.cls#OA\_versus\_normal\_repos |
| Upregulated in class | OA |
| GeneSet | GOBP\_CYTOSKELETON\_DEPENDENT\_CYTOKINESIS |
| Enrichment Score (ES) | 0.3252337 |
| Normalized Enrichment Score (NES) | 1.433972 |
| Nominal p-value | 0.017948719 |
| FDR q-value | 0.45065498 |
| FWER p-Value | 1.0 |
Table: GSEA Results Summary

  

Fig 1: Enrichment plot: GOBP\_CYTOSKELETON\_DEPENDENT\_CYTOKINESIS      
 Profile of the Running ES Score & Positions of GeneSet Members on the Rank Ordered List

  

| SYMBOL | TITLE | RANK IN GENE LIST | RANK METRIC SCORE | RUNNING ES | CORE ENRICHMENT || 1 | CHMP2B | charged multivesicular body protein 2B [Source:HGNC Symbol;Acc:HGNC:24537] | 129 | 0.876 | 0.0302 | Yes |
| 2 | DCTN3 | dynactin subunit 3 [Source:HGNC Symbol;Acc:HGNC:2713] | 136 | 0.871 | 0.0698 | Yes |
| 3 | CHMP4A | charged multivesicular body protein 4A [Source:HGNC Symbol;Acc:HGNC:20274] | 526 | 0.649 | 0.0693 | Yes |
| 4 | CHMP5 | charged multivesicular body protein 5 [Source:HGNC Symbol;Acc:HGNC:26942] | 556 | 0.638 | 0.0964 | Yes |
| 5 | WASHC5 | WASH complex subunit 5 [Source:HGNC Symbol;Acc:HGNC:28984] | 649 | 0.606 | 0.1170 | Yes |
| 6 | SEPTIN6 | septin 6 [Source:HGNC Symbol;Acc:HGNC:15848] | 826 | 0.562 | 0.1291 | Yes |
| 7 | CKAP2 | cytoskeleton associated protein 2 [Source:HGNC Symbol;Acc:HGNC:1990] | 1172 | 0.482 | 0.1244 | Yes |
| 8 | STAMBP | STAM binding protein [Source:HGNC Symbol;Acc:HGNC:16950] | 1227 | 0.471 | 0.1418 | Yes |
| 9 | ARL3 | ADP ribosylation factor like GTPase 3 [Source:HGNC Symbol;Acc:HGNC:694] | 1264 | 0.465 | 0.1604 | Yes |
| 10 | CHMP2A | charged multivesicular body protein 2A [Source:HGNC Symbol;Acc:HGNC:30216] | 1269 | 0.464 | 0.1814 | Yes |
| 11 | JTB | jumping translocation breakpoint [Source:HGNC Symbol;Acc:HGNC:6201] | 1346 | 0.451 | 0.1962 | Yes |
| 12 | STMN1 | stathmin 1 [Source:HGNC Symbol;Acc:HGNC:6510] | 1389 | 0.444 | 0.2133 | Yes |
| 13 | BBS4 | Bardet-Biedl syndrome 4 [Source:HGNC Symbol;Acc:HGNC:969] | 1404 | 0.442 | 0.2326 | Yes |
| 14 | AURKB | aurora kinase B [Source:HGNC Symbol;Acc:HGNC:11390] | 1407 | 0.442 | 0.2527 | Yes |
| 15 | SPAST | spastin [Source:HGNC Symbol;Acc:HGNC:11233] | 1502 | 0.429 | 0.2651 | Yes |
| 16 | SEPTIN2 | septin 2 [Source:HGNC Symbol;Acc:HGNC:7729] | 1528 | 0.424 | 0.2826 | Yes |
| 17 | TRIM36 | tripartite motif containing 36 [Source:HGNC Symbol;Acc:HGNC:16280] | 1907 | 0.372 | 0.2703 | Yes |
| 18 | EFHC2 | EF-hand domain containing 2 [Source:HGNC Symbol;Acc:HGNC:26233] | 1949 | 0.367 | 0.2840 | Yes |
| 19 | CHMP6 | charged multivesicular body protein 6 [Source:HGNC Symbol;Acc:HGNC:25675] | 2140 | 0.345 | 0.2850 | Yes |
| 20 | TTC19 | tetratricopeptide repeat domain 19 [Source:HGNC Symbol;Acc:HGNC:26006] | 2394 | 0.315 | 0.2798 | Yes |
| 21 | RASA1 | RAS p21 protein activator 1 [Source:HGNC Symbol;Acc:HGNC:9871] | 2506 | 0.302 | 0.2850 | Yes |
| 22 | RHOC | ras homolog family member C [Source:HGNC Symbol;Acc:HGNC:669] | 2694 | 0.280 | 0.2833 | Yes |
| 23 | KIF20B | kinesin family member 20B [Source:HGNC Symbol;Acc:HGNC:7212] | 2714 | 0.278 | 0.2946 | Yes |
| 24 | CHMP3 | charged multivesicular body protein 3 [Source:HGNC Symbol;Acc:HGNC:29865] | 2733 | 0.277 | 0.3059 | Yes |
| 25 | VPS4B | vacuolar protein sorting 4 homolog B [Source:HGNC Symbol;Acc:HGNC:10895] | 2783 | 0.271 | 0.3146 | Yes |
| 26 | SEPTIN10 | septin 10 [Source:HGNC Symbol;Acc:HGNC:14349] | 2806 | 0.269 | 0.3252 | Yes |
| 27 | ESPL1 | "extra spindle pole bodies like 1, separase [Source:HGNC Symbol;Acc:HGNC:16856]" | 3122 | 0.238 | 0.3116 | No |
| 28 | SON | SON DNA and RNA binding protein [Source:HGNC Symbol;Acc:HGNC:11183] | 3374 | 0.214 | 0.3019 | No |
| 29 | SEPTIN7 | septin 7 [Source:HGNC Symbol;Acc:HGNC:1717] | 3396 | 0.212 | 0.3100 | No |
| 30 | INCENP | inner centromere protein [Source:HGNC Symbol;Acc:HGNC:6058] | 3533 | 0.199 | 0.3085 | No |
| 31 | MAP9 | microtubule associated protein 9 [Source:HGNC Symbol;Acc:HGNC:26118] | 3535 | 0.199 | 0.3176 | No |
| 32 | PLK1 | polo like kinase 1 [Source:HGNC Symbol;Acc:HGNC:9077] | 3556 | 0.198 | 0.3251 | No |
| 33 | EFHC1 | EF-hand domain containing 1 [Source:HGNC Symbol;Acc:HGNC:16406] | 3778 | 0.179 | 0.3161 | No |
| 34 | ANK3 | ankyrin 3 [Source:HGNC Symbol;Acc:HGNC:494] | 3921 | 0.163 | 0.3125 | No |
| 35 | SEPTIN8 | septin 8 [Source:HGNC Symbol;Acc:HGNC:16511] | 4131 | 0.143 | 0.3028 | No |
| 36 | MYH10 | myosin heavy chain 10 [Source:HGNC Symbol;Acc:HGNC:7568] | 4169 | 0.141 | 0.3064 | No |
| 37 | NOX5 | NADPH oxidase 5 [Source:HGNC Symbol;Acc:HGNC:14874] | 4263 | 0.131 | 0.3052 | No |
| 38 | ALKBH4 | "alkB homolog 4, lysine demethylase [Source:HGNC Symbol;Acc:HGNC:21900]" | 4264 | 0.131 | 0.3112 | No |
| 39 | ZFYVE26 | zinc finger FYVE-type containing 26 [Source:HGNC Symbol;Acc:HGNC:20761] | 4366 | 0.123 | 0.3090 | No |
| 40 | UNC119 | unc-119 lipid binding chaperone [Source:HGNC Symbol;Acc:HGNC:12565] | 4617 | 0.103 | 0.2942 | No |
| 41 | PDCD6IP | programmed cell death 6 interacting protein [Source:HGNC Symbol;Acc:HGNC:8766] | 4956 | 0.076 | 0.2714 | No |
| 42 | IST1 | IST1 factor associated with ESCRT-III [Source:HGNC Symbol;Acc:HGNC:28977] | 5370 | 0.043 | 0.2411 | No |
| 43 | KIF23 | kinesin family member 23 [Source:HGNC Symbol;Acc:HGNC:6392] | 5400 | 0.040 | 0.2407 | No |
| 44 | CEP55 | centrosomal protein 55 [Source:HGNC Symbol;Acc:HGNC:1161] | 5787 | 0.009 | 0.2110 | No |
| 45 | POLDIP2 | DNA polymerase delta interacting protein 2 [Source:HGNC Symbol;Acc:HGNC:23781] | 5990 | -0.004 | 0.1955 | No |
| 46 | NUSAP1 | nucleolar and spindle associated protein 1 [Source:HGNC Symbol;Acc:HGNC:18538] | 6022 | -0.006 | 0.1933 | No |
| 47 | KIF20A | kinesin family member 20A [Source:HGNC Symbol;Acc:HGNC:9787] | 6457 | -0.039 | 0.1613 | No |
| 48 | SEPTIN11 | septin 11 [Source:HGNC Symbol;Acc:HGNC:25589] | 6591 | -0.049 | 0.1532 | No |
| 49 | NUP62 | nucleoporin 62 [Source:HGNC Symbol;Acc:HGNC:8066] | 6800 | -0.063 | 0.1398 | No |
| 50 | ACTR3 | actin related protein 3 [Source:HGNC Symbol;Acc:HGNC:170] | 7013 | -0.081 | 0.1270 | No |
| 51 | CUL7 | cullin 7 [Source:HGNC Symbol;Acc:HGNC:21024] | 7105 | -0.088 | 0.1240 | No |
| 52 | ARF1 | ADP ribosylation factor 1 [Source:HGNC Symbol;Acc:HGNC:652] | 7152 | -0.092 | 0.1246 | No |
| 53 | RACGAP1 | Rac GTPase activating protein 1 [Source:HGNC Symbol;Acc:HGNC:9804] | 7269 | -0.100 | 0.1202 | No |
| 54 | CHMP1B | charged multivesicular body protein 1B [Source:HGNC Symbol;Acc:HGNC:24287] | 7339 | -0.105 | 0.1197 | No |
| 55 | KIF4A | kinesin family member 4A [Source:HGNC Symbol;Acc:HGNC:13339] | 7480 | -0.117 | 0.1141 | No |
| 56 | ACTR2 | actin related protein 2 [Source:HGNC Symbol;Acc:HGNC:169] | 7498 | -0.118 | 0.1182 | No |
| 57 | SHCBP1L | SHC binding and spindle associated 1 like [Source:HGNC Symbol;Acc:HGNC:16788] | 7644 | -0.130 | 0.1129 | No |
| 58 | RAB35 | "RAB35, member RAS oncogene family [Source:HGNC Symbol;Acc:HGNC:9774]" | 7677 | -0.133 | 0.1165 | No |
| 59 | USP8 | ubiquitin specific peptidase 8 [Source:HGNC Symbol;Acc:HGNC:12631] | 8031 | -0.158 | 0.0962 | No |
| 60 | SEPTIN4 | septin 4 [Source:HGNC Symbol;Acc:HGNC:9165] | 8216 | -0.173 | 0.0898 | No |
| 61 | SPTBN1 | "spectrin beta, non-erythrocytic 1 [Source:HGNC Symbol;Acc:HGNC:11275]" | 8335 | -0.181 | 0.0889 | No |
| 62 | RHOA | ras homolog family member A [Source:HGNC Symbol;Acc:HGNC:667] | 8601 | -0.201 | 0.0775 | No |
| 63 | ECT2 | epithelial cell transforming 2 [Source:HGNC Symbol;Acc:HGNC:3155] | 8647 | -0.204 | 0.0834 | No |
| 64 | CENPA | centromere protein A [Source:HGNC Symbol;Acc:HGNC:1851] | 9311 | -0.256 | 0.0435 | No |
| 65 | SEPTIN9 | septin 9 [Source:HGNC Symbol;Acc:HGNC:7323] | 9336 | -0.258 | 0.0535 | No |
| 66 | CIT | citron rho-interacting serine/threonine kinase [Source:HGNC Symbol;Acc:HGNC:1985] | 9356 | -0.260 | 0.0639 | No |
| 67 | CHMP7 | charged multivesicular body protein 7 [Source:HGNC Symbol;Acc:HGNC:28439] | 9723 | -0.294 | 0.0489 | No |
| 68 | SEPTIN5 | septin 5 [Source:HGNC Symbol;Acc:HGNC:9164] | 10221 | -0.343 | 0.0259 | No |
| 69 | APC | APC regulator of WNT signaling pathway [Source:HGNC Symbol;Acc:HGNC:583] | 10440 | -0.366 | 0.0257 | No |
| 70 | ROCK2 | Rho associated coiled-coil containing protein kinase 2 [Source:HGNC Symbol;Acc:HGNC:10252] | 10648 | -0.388 | 0.0274 | No |
| 71 | VPS4A | vacuolar protein sorting 4 homolog A [Source:HGNC Symbol;Acc:HGNC:13488] | 10768 | -0.401 | 0.0366 | No |
| 72 | PLEC | plectin [Source:HGNC Symbol;Acc:HGNC:9069] | 10799 | -0.405 | 0.0529 | No |
| 73 | BIN3 | bridging integrator 3 [Source:HGNC Symbol;Acc:HGNC:1054] | 11043 | -0.436 | 0.0540 | No |
| 74 | CHMP1A | charged multivesicular body protein 1A [Source:HGNC Symbol;Acc:HGNC:8740] | 11401 | -0.487 | 0.0485 | No |
| 75 | ROCK1 | Rho associated coiled-coil containing protein kinase 1 [Source:HGNC Symbol;Acc:HGNC:10251] | 12046 | -0.605 | 0.0262 | No |
| 76 | RHOB | ras homolog family member B [Source:HGNC Symbol;Acc:HGNC:668] | 12713 | -0.878 | 0.0146 | No |
Table: GSEA details [plain text format]

  

Fig 2: GOBP\_CYTOSKELETON\_DEPENDENT\_CYTOKINESIS      
 Blue-Pink O' Gram in the Space of the Analyzed GeneSet

  

Fig 3: GOBP\_CYTOSKELETON\_DEPENDENT\_CYTOKINESIS: Random ES distribution      
 Gene set null distribution of ES for **GOBP\_CYTOSKELETON\_DEPENDENT\_CYTOKINESIS**
